# ^18^F-FDG PET/CT revealed primary malignant giant cell tumor of the sacrum: a case report

**DOI:** 10.3389/fmed.2025.1653522

**Published:** 2025-08-15

**Authors:** Zelong Feng, Ronghua Yu, Xianwen Hu

**Affiliations:** Department of Nuclear Medicine, Affiliated Hospital of Zunyi Medical University, Zunyi, China

**Keywords:** malignant giant cell tumor of bone, sacrum, 18F-FDG, PET/CT, MGCTB

## Abstract

Primary malignant giant cell tumor of bone (PMGCTB), which is usually confirmed to contain a high-grade sarcomatous component at the time of initial diagnosis, accounts for 1.6% of giant cell tumors of bone (GCTB). PMGCTB usually occurs in the epiphysis of long bones, which is similar to GCTB, and only 1.4–9.4% of GCTB occurs in the spine. PMGCTB in the spine is extremely rare. Herein, we present the case of a 46-year-old man who came to the hospital seeking medical help for lumbosacral pain. Computed tomography (CT) was performed because the clinician suspected that the patient had a herniated disk, and the results showed that the fifth lumbar vertebrae to the second sacral vertebrae showed bone destruction, accompanied by soft tissue tumors near the vertebrae, some of which protruded into the spinal canal and sacral canal. Magnetic resonance imaging (MRI) revealed that the lesion demonstrates an isointense signal on T1-weighted imaging (T1WI), a mixed hyperintense signal on T2-weighted imaging (T2WI), and obvious enhancement on contrast-enhanced T1WI. Fluorine-18 fluorodeoxyglucose (^18^F-FDG) positron emission tomography (PET)/CT imaging showed increased ^18^F-FDG uptake in the lesion. Subsequently, the patient underwent CT-guided biopsy and was diagnosed with PMGCTB by pathology. Because of the poor prognosis of PMGCTB, early diagnosis is essential for the rational treatment of PMGCTB. In the current study, we will review the relevant literature and discuss the clinical, imaging, pathological characteristics, and differential diagnosis of the relatively rare disease.

## Introduction

Malignant giant cell tumor of bone (MGCTB) is a type of tumor that combines conventional giant cell tumor and sarcomatous components (1–3). The World Health Organization (WHO) used the term “malignancy in GCTB” to describe MGCTB and subdivided it into either primary or secondary (4). The former refers to the presence of concurrent high-grade sarcomas at the time of initial diagnosis. In contrast, the latter refers to the presence of high-grade sarcomas at the same site after diagnosis of giant cell tumor of bone and treatment (5). MGCTB accounts for 4% of all GCTB cases (6). PMGCTB accounts for only 1.6%, which is considered extremely rare (1). The etiology of the disease is still unknown, but SMGCTB is associated with surgical irritation and radiation exposure (7, 8). The occurrence of MGCTB is associated with chromosomal abnormalities. Chromosome instability, heteroploidy, and centrosome aberration may be important factors for the malignant transformation of GCTB (9, 10). MGCTB is more common in women, with the majority of cases occurring in individuals aged 20–44 years (11). The distribution of MGCTB locations is similar to that of GCTB, with a predilection for the metaphysis of the long bones and a predominance in the knee, with only 1.4–9.4% of GCTB arising in the spine (12). Herein, we present the diagnosis and treatment of a 46-year-old patient with PMGCTB, focusing on the fluorine-18 fluorodeoxyglucose (^18^F-FDG) positron emission tomography (PET)/computed tomography (CT) to increase the understanding of this relatively rare tumor.

## Case presentation

**Figure 1:**
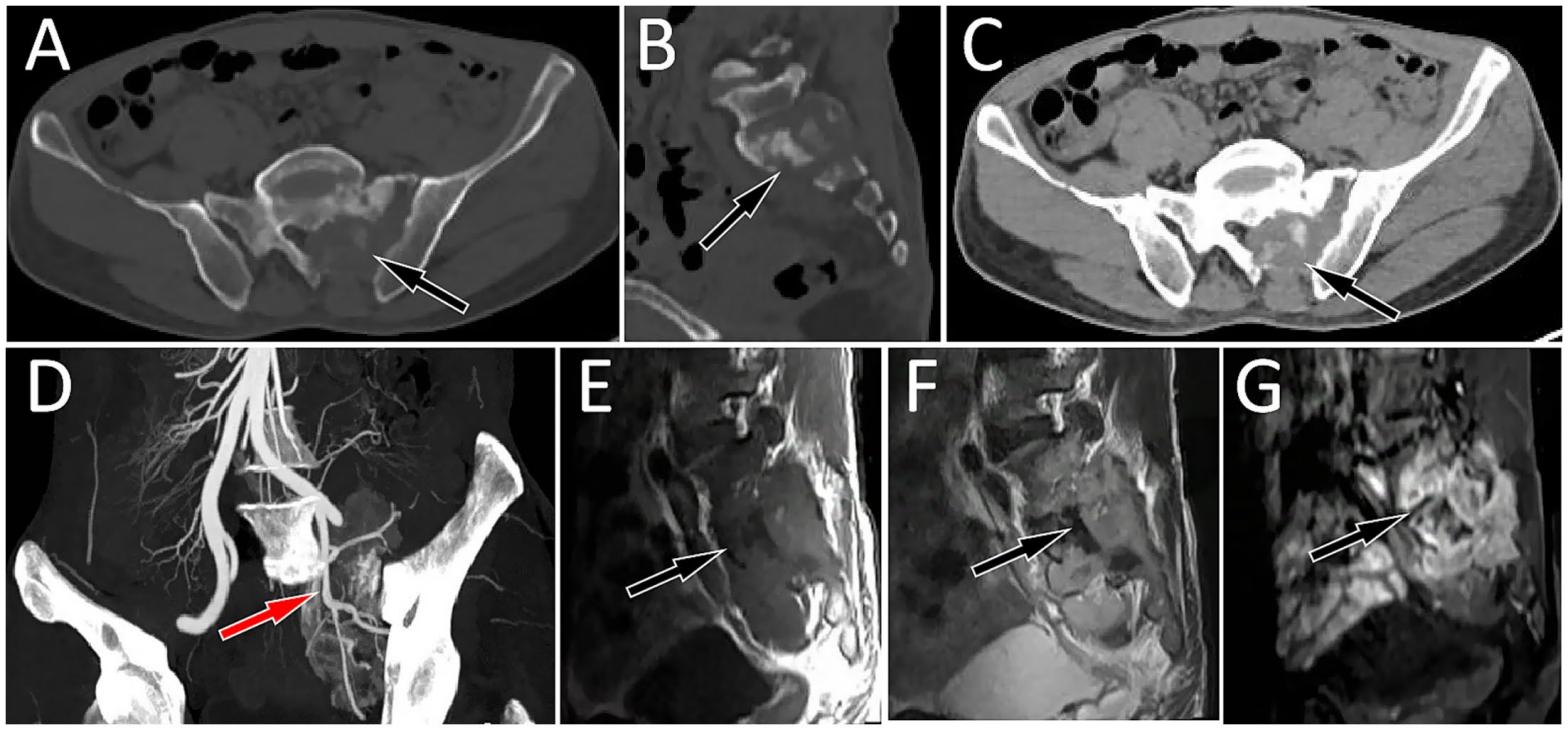
Axial CT bone window of the lumbosacral region shows a low-density bone destruction area in the left part of the sacrum and in the left appendage area (**A**, arrow). The sagittal view shows a bone sclerosis rim (**B**, arrow) in the destroyed bone. CT soft tissue window shows a soft tissue mass formation in the bone destruction area (**C**, arrow). **(D)** CT angiography revealed that the mass was supplied by the branches of the left internal iliac artery (arrow). Magnetic resonance imaging (MRI) shows that the abovementioned lesion presents as an isointense signal on T1-weighted imaging (T1WI, **E**) and a slightly hyperintense signal on T2-weighted imaging (T2WI, **F**) and shows significant enhancement on contrast-enhanced T1WI **(G)**.

**Figure 2:**
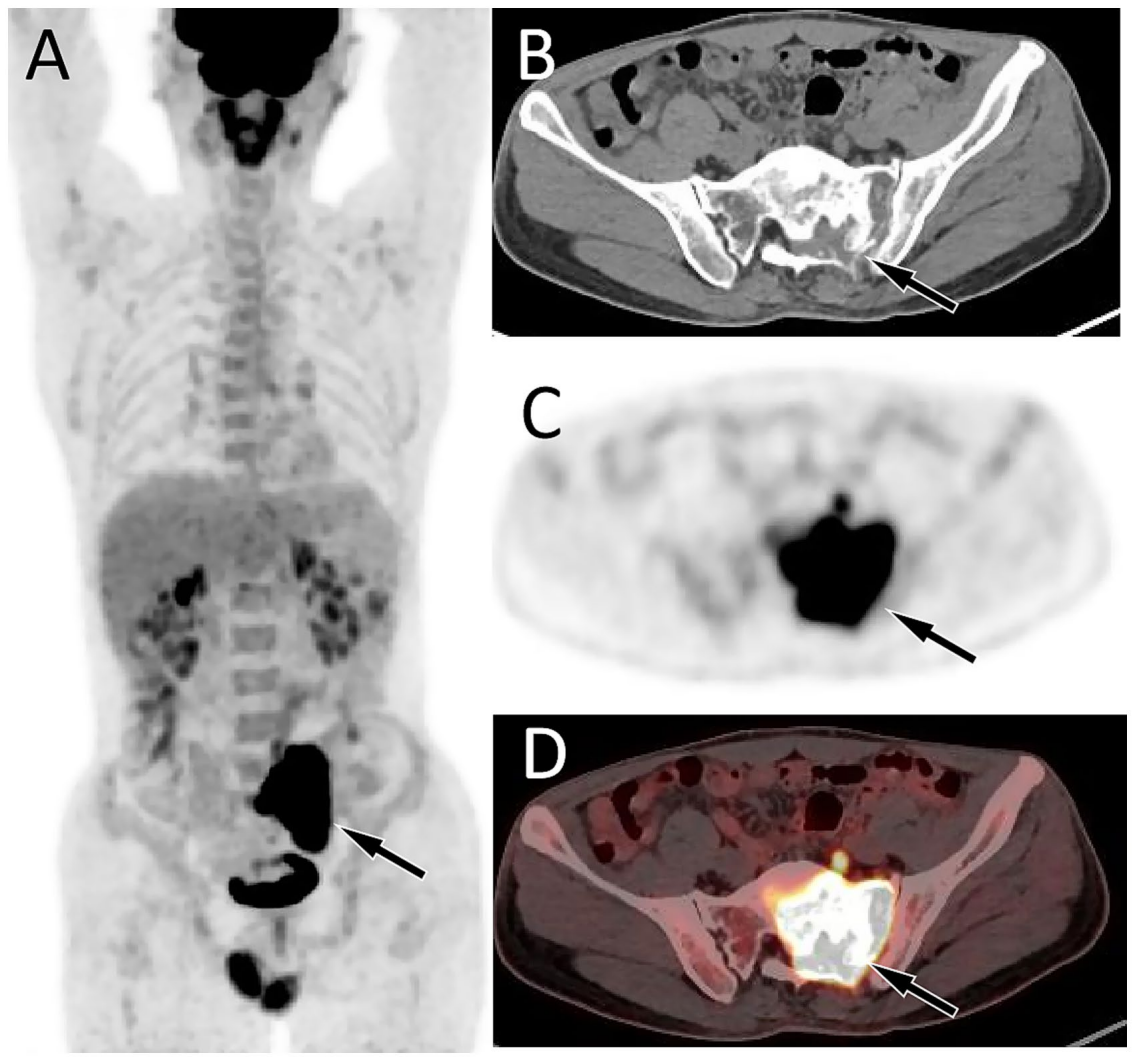
Fluorine-18 fluorodeoxyglucose (^18^F-FDG) positron emission tomography (PET)/CT imaging of the patient. The maximum intensity projection (MIP, **A**) showed an obviously increased ^18^F-FDG uptake in the central region of the pelvis (arrow). Axial CT **(B)** reveals the lesion localized in the left aspect of the sacrum and left appendicular region (arrow). The corresponding lesion had obviously increased ^18^F-FDG uptake on axial PET **(C)** and PET/CT fusion **(D)**, with a maximum standardized uptake value (SUVmax) of 33.6.

**Figure 3:**
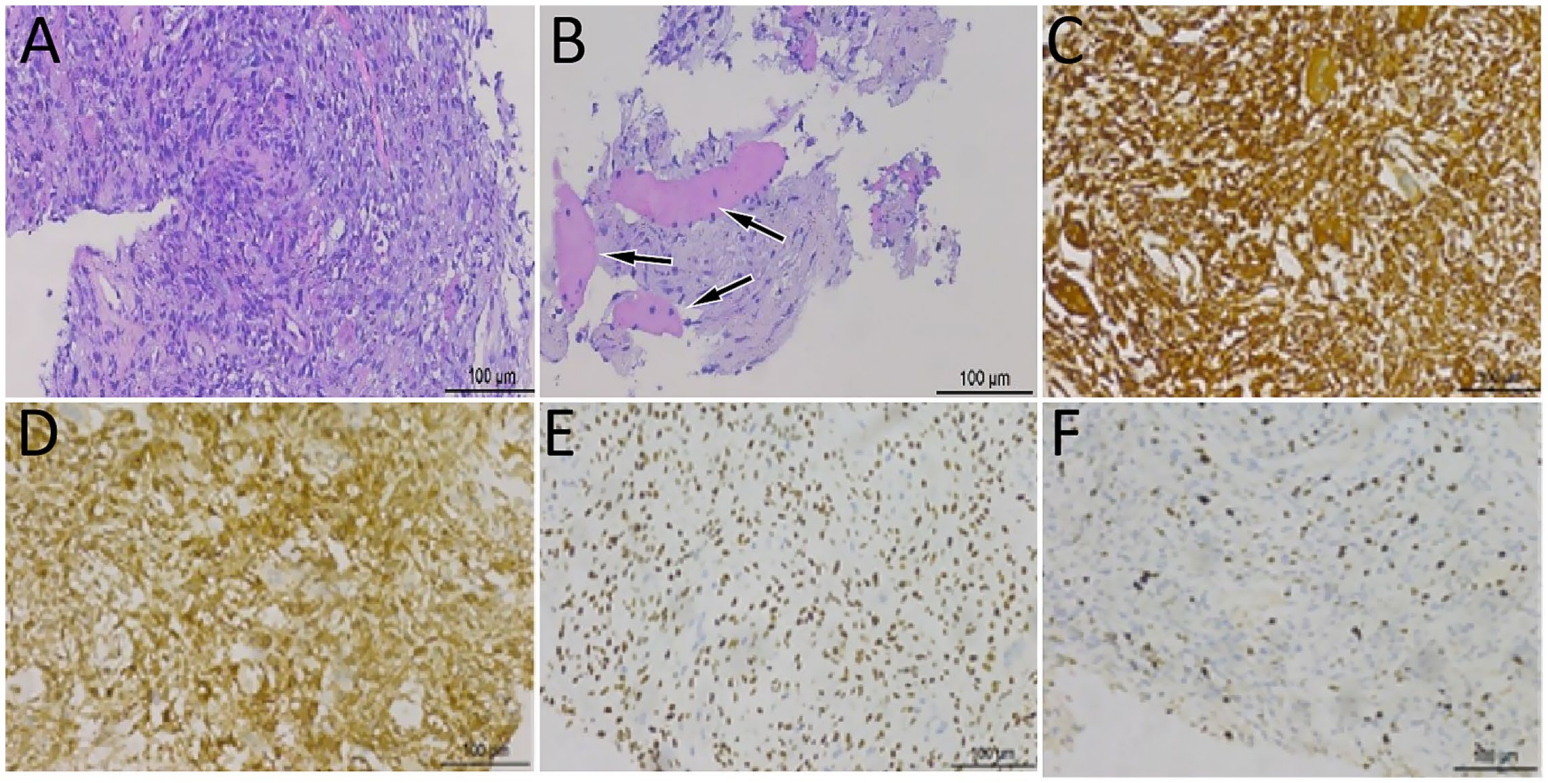
Hematoxylin–eosin staining showed that spindle cells and osteoclast-like giant cells were mixed in the mass **(A)**, accompanied by high-grade sarcoma components (**B**, arrows). Immunohistochemical analysis showed that the tumor cells positively expressed vimentin **(C)**, H3.3G34 **(D)**, SATB2 **(E)**, and osteoclast-like giant cell CD68 **(F)**.

A 46-year-old man without any previous medical history presented to our hospital with lumbosacral pain for 4 months. Physical examination showed that the patient suffered from pain in the waist, and there were no positive signs in the rest of the body. The patient’s hemogram and tumor marker values were all within the normal reference range. Lumbar and sacral CT showed that the patient’s fifth lumbar vertebrae to the second sacral vertebrae and its appendages had low-density bone destruction, accompanied by a paravertebral soft tissue mass (6.7 cm × 5.3 cm × 8.2 cm), and partial protrusion into the vertebral and sacral canals; CT angiography showed that the mass was supplied by the left internal iliac artery (as shown in Figure 1). Magnetic resonance imaging (MRI) shows that the aforementioned lesion presents as an isointense signal on T1-weighted imaging (T1WI) and slightly hyperintense signal on T2-weighted imaging (T2WI) and shows significant enhancement on contrast-enhanced T1WI. Subsequently, PET/CT was performed to evaluate the tumor’s nature and staging. The results demonstrated markedly increased ^18^F-FDG uptake in the lesion spanning the fifth lumbar vertebra to the second sacral vertebra, with a maximum standard uptake value (SUVmax) of 33.6 (Figure 2). Based on the patient’s abovementioned imaging findings, it is highly likely that the patient has a malignant spinal tumor. The patient underwent CT-guided biopsy. Hematoxylin–eosin staining showed that spindle cells and osteoclast-like giant cells were mixed in the mass, which was a typical manifestation of GCTB. Moreover, there is a small amount of high-grade sarcomatous elements, with localized tumor invasion of skeletal muscle at the margin (as shown in Figure 3). Immunohistochemical findings showed positive expression of vimentin, H3.3G34, SATB2, osteoclast-like giant cell CD68, SMA, skeletal muscle desmin, and Ki-67, with a positivity rate of approximately 10%. In contrast, a negative expression was observed for CD1a, S100, ALK, CD163, CK, and P63. Based on these pathological and immunohistochemical findings, the patient was diagnosed with PMGCTB from the fifth lumbar vertebrae to the second sacral vertebrae. After the diagnosis was confirmed, the patient received three courses of denosumab treatment but showed a poor response. Two months later, we were informed that the patient had passed away, and we have not had more information about the treatment and outcome.

## Discussion

MGCTB is mainly characterized by osteolytic bone destruction, and patients often come to the hospital for medical help because of bone pain. The diagnosis of MGCTB is mainly based on biopsy. The presence of high-grade sarcoma within a typical giant cell tumor of bone is mandatory for the diagnosis of MGCTB, which is essentially a dedifferentiated sarcoma occurring from a giant cell tumor (13). The high-grade sarcoma components of MGCTB include osteosarcoma, undifferentiated sarcoma, and fibrosarcoma. Among them, osteosarcoma is the most common one (3). In this case, in addition to the typical giant cell tumor of bone, high-grade sarcoma components were present, along with local tumor infiltration of skeletal muscle at the edge, which was consistent with the diagnosis of MGCTB. However, PMGCTB may be initially missed because of sampling errors at biopsy, too small sample, or overly conservative pathologist’s judgment, with the primary malignancy being detected retrospectively only when the specimen is reevaluated (12).

Imaging examinations are complementary to pathological assessment in the diagnosis of MGCTB, and common imaging examinations include CT, MRI, and PET/CT. On CT, MGCTB mainly grows eccentrically and manifests osteolytic bone destruction, with an irregular bone crest in internal shape and incomplete bone encasement in the periphery, and the bone shell is misaligned, and the boundary is unclear (14). On T1WI, the area of bone destruction is isointense, and contrast-enhanced T1WI scans showed obvious inhomogeneous enhancement; on T2WI, the high signal of the lesion was displayed (15). As the imaging presentation of this disease is not specific, it is difficult to make a specific diagnosis using conventional imaging. PET/CT is of great value in revealing tumor metabolism and plays a significant role in the detection, staging, and treatment of many sarcomas and cancers (16). GCTB usually shows hypermetabolism on ^18^F-FDG PET/CT scans. Muheremu et al. (17) identified 20 patients with GCTB, with an average SUVmax of 9.2. The average SUVmax of GCTB in the pelvis and spine is higher than that in the limbs, at approximately 10.4. This might be the result of the overexpression of GLUT-1 and hexokinase-2 in macrophages and giant cells in tumors (18). Before our study, only two studies (19, 20) with a total of three MGCTB patients’ PET/CT findings were described, with SUVmax values ranging from 21.0 to 31.7, as detailed in Table 1. The SUVmax of this case was 33.6, and the metabolism was significantly higher than that of GCTB. Presumably, as in most other malignancies, both the percentage of tumor vessels to tumor volume and the rate of tumor glycolysis increased, thus showing a significant increase in ^18^F-FDG uptake. Therefore, it is important to consider the diagnosis of GCTB and soft tissue sarcoma in isolated musculoskeletal lesions with high ^18^F-FDG uptake (16). However, the ^18^F-FDG PET/CT findings of malignant giant cell tumor of bone are rarely reported, and more samples are needed to prove its effectiveness.

**Table 1:** The clinical and PET/CT features of patients with malignant giant cell tumor of bone.

Case	Author/year	Gender/age	Location	MD (cm)	PET/CT findings					Management	Follow-up (months)
					Density	Bone cortex	Growth pattern	Sclerotic rim	SUVmax		
1 (19)	Vari S /2022	F/22	Right femur	6.9	Low	Integrity	Longitudinal axis	Yes	21.0	Surgery+chemotherapy	53/dead
2 (19)		M/48	Right tibia	10.0	Low	Interruption	Longitudinal axis	No	26.5	Surgery+chemotherapy	51/alive with disease
3 (20)	Donigian S/2022	M/24	Right femur	6.8	NA	NA	NA	NA	31.7	Surgery+chemotherapy	7/alive with disease
3	Present case	M/46	Sacrum	8.2	Low	Interruption	Longitudinal axis	No	33.6	Chemotherapy	5/dead

M, male; F, female; MD, maximum diameter; CT, computed tomography; PET, positron emission tomography; NA, not applicable.

Spinal MGCTB should be differentiated from GCTB, chordoma, and metastatic bone tumor. GCTB is an intermediate-type tumor that commonly occurs in young adults. On CT, it presents as eccentric and expansive bony destruction with sharply demarcated sclerosis, an orderly arrangement of the internal bony ridges, and a rare periosteal reaction (14, 21). On PET/CT, the lesions are metabolically active, with a mean SUVmax of 9.2, while it was significantly lower than the SUVmax of MGCTB. Chordomas have a predilection for the sacrococcygeal region, often have osteolytic bone destruction radiologically, and tend to form soft-tissue masses outside the spinal canal (22). The mean SUVmax before treatment is 5.1 (23). Metastatic bone tumors are common in middle-aged and elderly people and usually have primary tumors, mainly present as osteolytic destruction of the posterior parts of the vertebral bodies, with poorly defined borders, frequent involvement of the pedicles, and formation of paraspinal soft tissue masses (24). These several categories of tumors have overlapping clinical and imaging features, making accurate diagnosis challenging. In addition, mutation detection is being evaluated as an adjunct to the diagnosis of GCTB, and H3F3A mutations may help distinguish GCTB from giant cell-rich sarcomas (25).

Due to the rarity of MGCTB, there is no consensus on its treatment recommendations, and the protocol includes surgery alone or surgery combined with chemotherapy or radiotherapy (26). Surgical resection is the treatment of choice, and adequate surgical margins are believed to be associated with reduced recurrence rates in MGCTB (27). However, spinal MGCTB is difficult to resect surgically, and radiotherapy and chemotherapy are used as adjunctive therapies for MGCTB, but their positive effects on recurrence and overall survival remain controversial (11). It has been suggested that MGCTB is radiation-resistant, and malignant transformation occurs after radiotherapy (28, 29). However, studies have found that chemotherapy can be applied to sites where therapeutic surgery is not feasible, such as the spine or sacrum (30). Denosumab, a human monoclonal antibody that inhibits RANKL, has emerged as a novel treatment option for locally advanced GCTB (14). After denosumab treatment, large areas of mixed osteogenesis are frequently observed in patients with GCTB, with multiple osteosclerotic septations within the tumor and an intact sclerotic bone shell at the edges of the soft-tissue mass, making surgical resection possible (31). The same radiographic changes were observed in this patient with PMGCTB after 3 cycles of denosumab chemotherapy. The prognosis of MGCTB was generally poor. The 5-year survival rates of PMGCTB and SMGCTB were 56.2 and 40%, respectively (27). This current patient died 2 months after chemotherapy due to tumor metastasis, further demonstrating the highly malignant nature of the disease and its poor prognosis.

## Conclusion

Spinal MGCTB is rare, and an accurate diagnosis is challenging. The diagnosis of MGCTB should be combined with clinical manifestations, imaging examinations, and pathology to render a more comprehensive judgment. Our case suggests that ^18^F-FDG PET/CT is helpful in the differential diagnosis of MGCTB; however, this needs to be confirmed in a larger number of cases in the future.

## Data Availability

The original contributions presented in the study are included in the article/supplementary material, further inquiries can be directed to the corresponding authors.
